# Supplementary material for: First Characterization of Human Dermal Fibroblasts Showing a Decreased Xylosyltransferase-I Expression Induced by the CRISPR/Cas9 System
Source: Int J Mol Sci. 2022 May 2;23(9):5045. doi: 10.3390/ijms23095045 (PMC9100032; doi:10.3390/ijms23095045)
Supplement: Supplementary file 1 [file ijms-23-05045-s001.zip › ijms-1693875-supplementary.pdf]

Supplement

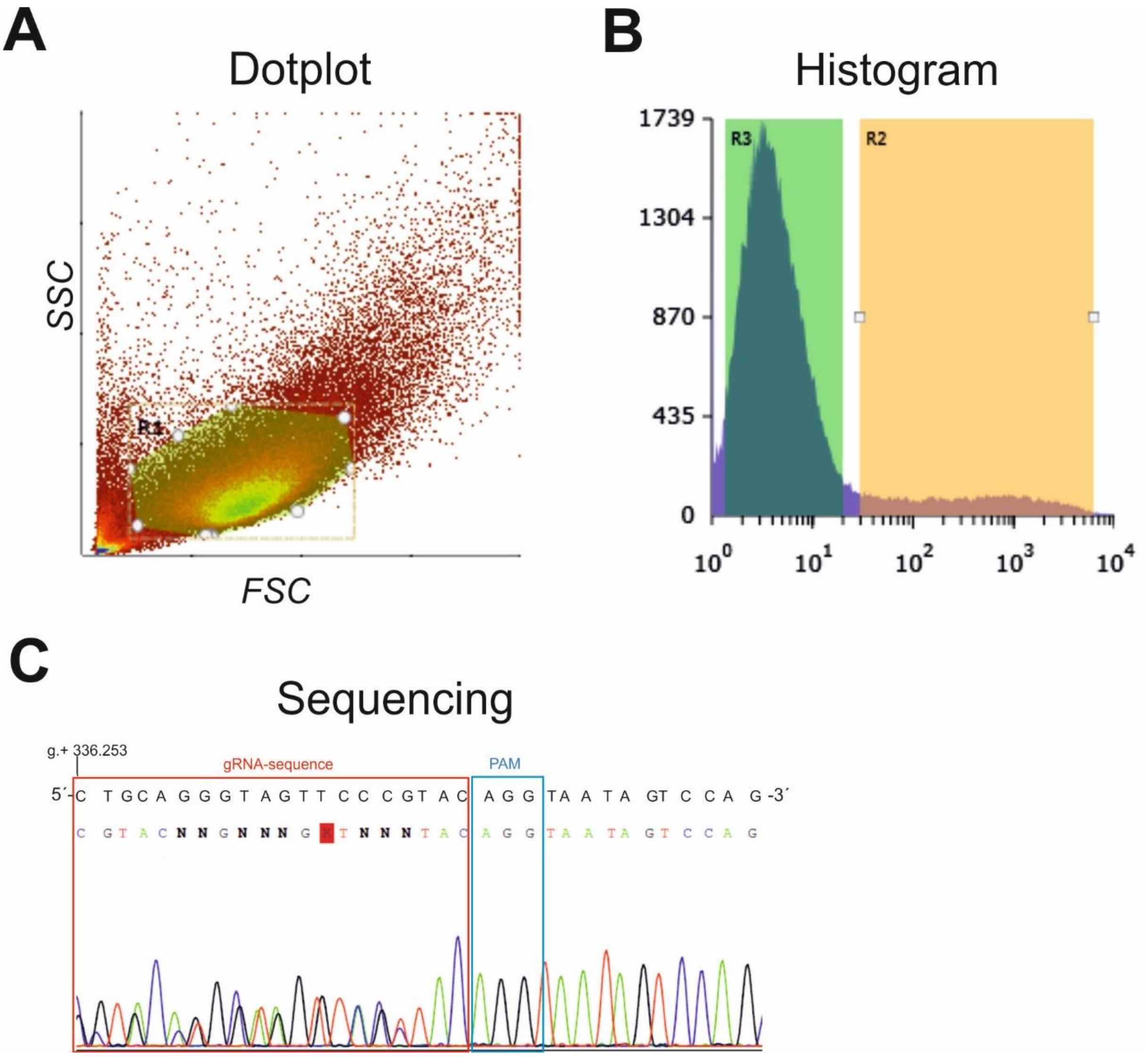

Figure S1: Cell-sorting and sequencing analysis of *XYLT*$^{-/-}$ fibroblasts. Cells were transfected with 1.5 µg of the vector PX458. The vector inter alia encoded GFP, allowing fluorescence-based separation of transfected and non- transfected cells using FACS technology. The dotplot diagram (A) shows the determined side-scatter (SSC, measure of granularity) plotted against the forward-scatter (FSC, measure of cell-size). The resulting histogram (B) shows the resulting fluorescence distribution of the selected cell-population. Cells showing a high fluorescence intensity were isolated (B, yellow box). In total, 10.715 cells were isolated. To examine the success of gene editing, Sanger sequencing of the isolated DNA was performed (C). The sequence shown starts at base g.+ 336.253 (genbank accession ID: NG_015843.2) in the genomic DNA isolated from M1FS cells. Highlighted are the gRNA sequence complementary binding within exon 9 of the *XYLT1* gene (red box) and the PAM sequence essential for Cas9 binding (blue box).
